# The Multidisciplinary Approach to GLP-1 RA and SGLT2 Inhibitors in Cardiometabolic Care: A New Era for Patients with Diabetes and Heart Disease

**DOI:** 10.3390/jcm14144834

**Published:** 2025-07-08

**Authors:** Fabiana Lucà, Maurizio Giuseppe Abrignani

**Affiliations:** 1 Department of Cardiology, Grande Ospedale Metropolitano (GOM) of Reggio Calabria, Bianchi Melacrino Morelli Hospital, 89129 Reggio Calabria, Italy; 2 Department of Cardiology, Paolo Borsellino Hospital, 91025 Marsala, Italy; maur.abri60@gmail.com

Editorial

Cardiovascular diseases (CVDs) represent a prominent clinical challenge, particularly among patients with chronic comorbidities such as type 2 diabetes mellitus (T2DM) and chronic kidney disease (CKD) [1,2]. These comorbidities complicate patient management due to the need for multiple pharmacological treatments and the increased risk of drug interactions that is associated with them [3].

A practical approach to managing CVD thus requires the implementation of a well-coordinated and personalized care pathway targeted at delivering optimized treatment through collaboration among various healthcare professionals [3,4,5,6].

Team-based care involves multidisciplinary and multi-professional collaboration including healthcare professionals from various fields and community health workers such as nurses, dietitians, psychologists, and physiotherapists [7]. Physicians who manage patients with CVD must be updated on the latest developments in the full spectrum of heart failure (HF) [8,9,10], as well as coronary artery disease (CAD) [11,12,13,14] and other related conditions.

In this editorial, we provide an overview of the intersection of type 2 diabetes mellitus (T2DM) and cardiovascular disease (CVD), emphasizing the need for a strategic, multidisciplinary model of care, with a specific focus on the use of sodium-glucose cotransporter-2 inhibitors (SGLT2i) and glucagon-like peptide-1 receptor agonists (GLP-1 RAs). While numerous contributions in this Special Issue examine broader aspects of cardiovascular care [15,16,17,18,19,20], we focused on the role of GLP-1-RAs [21,22] and SGLT2i [23,24], representing a revolution in the management of patients with both T2DM and CVD [21,25].

Although initially limited to treating diabetic patients, impressive beneficial effects of CV have been observed, leading to an evolution in the therapeutic strategies that can be used in high-risk cardiometabolic patients [26,27]. However, regular use is required before becoming proficient in managing these drugs. A collaborative, multidisciplinary approach including specialists from various fields will allow us to address the complex interplay between T2DM and CVD holistically [3].

GLP-1 RAs and SGLT2i have expanded the boundaries of T2DM care by significantly reducing CV events and hospitalizations due to HF, as shown in landmark studies like LEADER [28], SUSTAIN-6 [29], and EMPA-REG OUTCOME [30].

Benefits for Atherosclerotic Cardiovascular Disease (ASCVD)

Beyond glycemic control, GLP-1 RAs have been shown to reduce body weight, improve lipid profiles, and lower blood pressure [29]. The SELECT [31] and FLOW [32] trials represent pivotal studies that have demonstrated the efficacy of GLP-1 RA in improving CV and renal outcomes. Specifically, SELECT provided evidence of CV benefits in individuals who were overweight or obese without DM or established atherosclerotic cardiovascular disease (ASCVD).

Benefits for Chronic Kidney Disease (CKD)

In contrast, FLOW confirmed the presence of renoprotective effects in patients with T2DM and CKD [31]. The SOUL trial has recently provided robust evidence supporting the CV effectiveness of oral semaglutide—the first GLP-1 RA available in an oral formulation—in individuals with T2DM and established ASCVD and/or CKD. The observed reduction in the incidence of major adverse CV events (MACEs), including myocardial infarction (MI) and coronary revascularisation, underscores the molecule’s inherent anti-atherosclerotic properties [33]. The reduction in MACEs observed with oral semaglutide occurred alongside the provision of standard-of-care therapies, with approximately 49% of trial participants receiving SGLT2i at some stage during the study. Notably, the CV benefits of semaglutide were evident irrespective of baseline SGLT2i use, indicating comparable efficacy in both individuals receiving SGLT2i therapy and those not undertaking it throughout the SOUL trial [34].

Benefits for Heart Failure (HF)

In contrast, SGLT2i effectively reduce HF risk, prevent renal progression, and confer survival benefits to patients [23]. These findings suggest that both drug classes are suitable for cardiometabolic patients who require comprehensive risk reduction beyond glucose management [35].

Benefits for Obesity

Obesity has emerged as a condition of growing significance in the field of cardiology, given its strong association with elevated risks of CVD and HFpEF [36,37]. Rather than being merely a contributing risk factor, obesity is now increasingly identified as a principal etiological driver of HFpEF [38]. Epidemiological data indicate that up to 80% of individuals diagnosed with HFpEF are affected by overweight or obesity— a prevalence more than double that observed in the general population [39,40]. Within this clinical framework, semaglutide has demonstrated potential as a therapeutic option not only for achieving weight reduction but also for enhancing health-related quality of life and physical functional status in patients with obesity-related HFpEF, regardless of the presence of T2DM, as evidenced by findings from the STEP-HFpEF [41] and STEP-HFpEF T2DM trials [42].

Integrated Findings

An integrated analysis of data from four randomized, placebo-controlled clinical trials—SELECT, FLOW, STEP-HFpEF, and STEP-HFpEF T2DM—included a total cohort of 22,282 participants, among whom 3743 individuals (16.8%) had a prior diagnosis of HFpEF. In this population, semaglutide treatment resulted in a statistically significant relative reduction of 31% in the composite outcome of CV death or worsening HF compared to that in the placebo group [43]. Based on the available evidence and the distinct mechanisms of action, these two classes of cardioprotective drugs could be clinically applied by utilizing the proposed treatment algorithm and interdisciplinary management tailored to the specific patient phenotype (Figure 1).

Managing patients with both T2D and CVD demands a nuanced approach that addresses not only hyperglycemia but also hypertension, dyslipidemia, obesity, and renal function. This level of complexity necessitates a collaborative framework among specialists, including cardiologists, endocrinologists, nephrologists, primary care physicians, pharmacists, dietitians, and diabetes educators. Each team member brings unique expertise, contributing to an integrated care model that improves patient outcomes.

For instance, cardiologists are increasingly aware of the benefits of GLP-1 RA and SGLT2i therapies in reducing CV events and HF risk. However, they may rely on endocrinologists for insights into these agents’ metabolic effects of these agents and optimal dosing. Meanwhile, nephrologists play a critical role in managing the renal implications of both drug classes, particularly SGLT2 i, which offer renal-protective effects crucial for patients with compromised kidney function [29]. In this context, multidisciplinary teamwork ensures that patients receive the most comprehensive and effective care.

While the advantages of a multidisciplinary approach are clear, implementing it presents several challenges. Key barriers include the need for coordinated communication among specialists, the alignment of treatment goals, and consistent patient education. To overcome these obstacles, institutions can establish cardiometabolic clinics that integrate multiple specialties in a single setting. These clinics foster seamless communication, allowing healthcare providers to develop unified treatment plans that consider the entire spectrum of the patient’s needs.

Additionally, electronic health records (EHRs) can facilitate information sharing among team members, enabling real-time patient progress updates to be communicated to relevant parties and ensuring that all parties are informed of therapeutic changes. EHR platforms also offer opportunities to flag potential drug–drug interactions, adjust dosing in response to renal function, and monitor patient adherence.

Education and training for healthcare professionals and patients are critical to the success of a multidisciplinary approach. Providers should receive training on the latest evidence regarding GLP-1 RA and SGLT2i therapies, including their cardiovascular and renal benefits, to confidently recommend these options within their specialties. On the other hand, patient education helps individuals understand the multifaceted nature of their treatment plans, fostering better adherence and engagement in their care journey [44].

As the evidence for GLP-1 RAs and SGLT2i continues to grow, a multidisciplinary model of care could set a new standard for managing patients with T2DM and CVD. This integrated approach improves individual health outcomes and reduces the burden of hospitalizations and complications associated with cardiometabolic diseases, making it a cost-effective strategy that benefits both patients and healthcare systems.

In the future, further integration of these therapies into cardiometabolic guidelines, along with continued research on their long-term effects, will likely strengthen the case for their widespread use. For now, healthcare teams are encouraged to embrace multidisciplinary collaboration, recognizing that the expertise of each team member contributes to a comprehensive, patient-centered approach that transforms the lives of those facing the dual challenge of diabetes and heart disease.

The convergence of diabetes and CVD is a complex clinical issue that demands innovative solutions [45]. GLP-1 RAs and SGLT2i offer a beacon of hope for patients by addressing both metabolic and CV risks. However, their optimal use requires a multidisciplinary team dedicated to providing holistic, coordinated care (Figure 2). By working together across specialties, healthcare providers can maximize the benefits of these therapies, reduce the incidence of cardiovascular events, and set a new standard for cardiometabolic care that truly puts patients at the center of their care journey.

## Figures and Tables

**Figure 1:**
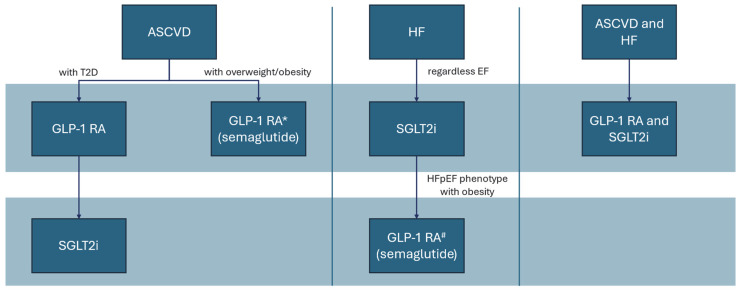
Proposed algorithm treatment choice depending on cardiovascular patient phenotype. GLP-1 RAs with proven benefits in ASCVD patients with overweight or obesity. #GLP-1 RAs with clinical benefit on HFpEF patients with obesity. Abbreviations: ASCVD: atherosclerotic cardiovascular disease; EF: ejection fraction; HF: heart failure; HFpEF: heart failure with preserved ejection fraction; GLP-1 RA: glucagon-like peptide-1 receptor agonists; SGLT2i, sodium-glucose co-transporter-2 inhibitors; T2D: type 2 diabetes.

**Figure 2:**
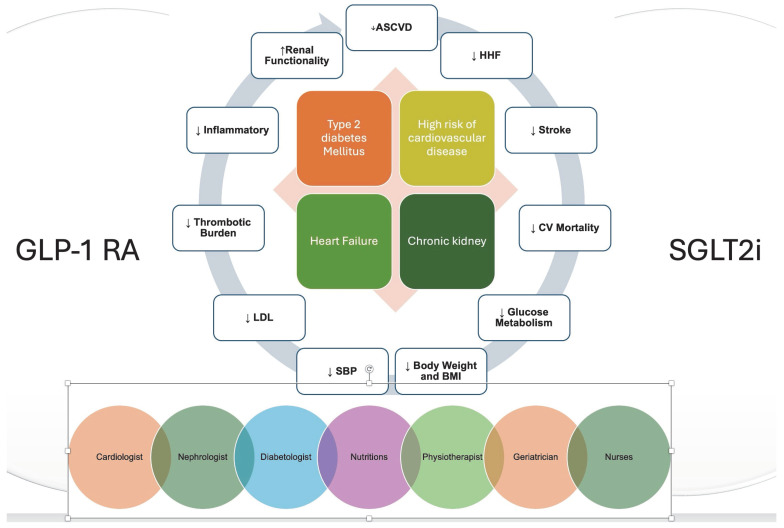
Multidisciplinary approach to cardiometabolic care. Abbreviations: GLP-1-RA: glucagon-like peptide 1 receptor agonists; SGLT2i: sodium-glucose cotransporter-2 inhibitors, HHF: hospitalization for heart failure; CV: cardiovascular; MACE: major adverse cardiovascular events; AMI: acute myocardial infarction; SBP: systolic blood pressure; LDL: low-density-lipoprotein cholesterol.
